# Role of Gpcpd1 in intestinal alpha-glycerophosphocholine metabolism and trimethylamine *N*-oxide production

**DOI:** 10.1016/j.jbc.2024.107965

**Published:** 2024-11-05

**Authors:** Siyi Chen, Shiho Inui, Rahmawati Aisyah, Ryoko Nakashima, Tatsuya Kawaguchi, Minori Hinomoto, Yoshiko Nakagawa, Tetsushi Sakuma, Yusuke Sotomaru, Noriyasu Ohshima, Thanutchaporn Kumrungsee, Takeshi Ohkubo, Takashi Yamamoto, Yutaka Miura, Takuya Suzuki, Noriyuki Yanaka

**Affiliations:** 1 Graduate School of Integrated Sciences for Life, Hiroshima University, Hiroshima, Japan; 2 Center for Animal Resources and Development (CARD), Kumamoto University, Kumamoto, Japan; 3 Natural Science Center for Basic Research and Development, Hiroshima University, Hiroshima, Japan; 4 Graduate School of Medicine, Gunma University, Gunma, Japan; 5 Faculty of Human Sciences, Sendai Shirayuri Women's College, Sendai, Japan; 6 Institute of Agriculture, Tokyo University of Agriculture and Technology, Tokyo, Japan

**Keywords:** glycerophosphocholine, trimethylamine *N*-oxide, choline, Gpcpd1, GDE5

## Abstract

Glycerophosphocholine (GPC) is an intracellular metabolite in phosphatidylcholine metabolism and has been studied for endogenous choline supply in cells. GPC, as a water-soluble supplement, has been expected to play a role in preventing brain disorders; however, recent studies have shown that intake of high levels of choline-containing compounds is related to trimethylamine *N*-oxide (TMAO) production in the liver, which is reportedly associated with the progression of atherosclerosis. In this study, we aimed to explore the mechanisms underlying the intestinal absorption and metabolism of GPC. Caco-2 cell monolayer experiments showed that exogenously added GPC was hydrolyzed to choline in the apical medium, and the resulting choline was transported into the Caco-2 cells and further to the basolateral medium. Subsequently, we focused on glycerophosphodiesterase 1 (Gpcpd1/GDE5), which hydrolyzes GPC to choline *in vitro* and is widely expressed in the gastrointestinal epithelium. Our results revealed that the Gpcpd1 protein was located not only in cells but also in the medium in which Caco-2 cells were cultured. *Gpcpd1* siRNA decreased the GPC-hydrolyzing activity both inside Caco-2 cells and in conditioned medium, suggesting the involvement of Gpcpd1 in luminal GPC metabolism. Finally, we generated intestinal epithelial–specific Gpcpd1-deficient mice and found that Gpcpd1 deletion in intestinal epithelial cells affected GPC metabolism in intestinal tissues and partially abolished the increase in blood TMAO levels induced by GPC administration. These observations demonstrate that Gpcpd1 triggers choline production from GPC in the intestinal lumen and is a key endogenous enzyme that regulates TMAO levels following GPC supplementation.

Choline is recognized as an essential nutrient for cell membrane and acetylcholine production and plays an important role in maintaining normal cell functions (1). Phosphatidylcholine (PC) is a phospholipid that makes up 45 to 55% of lipids in mammalian cell membranes and is also utilized for the formation of lipoproteins, such as chylomicrons and very low–density lipoproteins, which are essential for lipid transport (2, 3). Acetylcholine is an important neurotransmitter in cholinergic neurons and is involved in signaling related to brain functions such as memory and learning (4, 5). However, epidemiological studies have indicated that choline intake is insufficient in many countries; exogenous sources of choline derived from supplements are expected to meet choline requirements (6).

Glycerophosphocholine (GPC) is a well-known intermediate in PC metabolism, which is essential for eukaryotic cell membranes. In mammals, PC degradation mainly relies on a phospholipase pathway, phospholipase A_1_/phospholipase A_2_, and lysophospholipase pathway, which hydrolyzes PC into free fatty acids and GPC (Fig. 1) (7, 8). GPC is a major water-soluble form of stored choline and possibly hydrolyzed to choline by glycerophosphodiesterase (GDE/Gpcpd) family of enzymes (9, 10). The resulting choline is further utilized for PC biosynthesis (The Kennedy pathway) or oxidized to betaine, a methyl donor for the conversion of homocysteine to methionine in one-carbon metabolism. GPC is a globally used source of choline in supplements. Notably, recent studies have demonstrated that GPC supplementation improves cognition, behavior, and functional outcomes in neurodegenerative disorders in animal models and human clinical studies (11, 12, 13). The cognitive benefits associated with increased acetylcholine availability are more pronounced with supplementation with GPC than with other cholinergic precursors such as CDP-choline (14). The probable reason for this is that GPC administration can increase acetylcholine levels in the hippocampus, leading to significant enhancements in cognitive ability and memory in Alzheimer’s disease, stroke, and cerebral ischemic attacks as indicated by numerous studies (15). In intestinal tissues, GPC may be hydrolyzed to choline, which is subsequently oxidized to betaine (Fig. 1). Additionally, growing evidence suggests that choline-containing compounds can be metabolized by the intestinal microbiota into trimethylamine (TMA), which is subsequently absorbed through the intestine and further converted into trimethylamine *N*-oxide (TMAO) in the liver (Fig. 1). TMAO is currently being investigated for its pathological relationship with atherosclerosis progression (16, 17, 18, 19). The anaerobic degradation of choline in the intestine begins with a TMA-generating cleavage reaction, facilitated by the choline utilization gene (Cut) cluster. This includes a glycyl radical enzyme (GRE), choline TMA-lyase (CutC), and GRE activase (CutD), all of which contribute to TMA production (20, 21). Upon absorption *via* the intestinal epithelium, TMA is metabolized into TMAO by hepatic monooxygenases (22). Despite these advancements in our understanding of the benefits of GPC supplementation, the exact mechanism by which GPC is absorbed and metabolized in the digestive tract has not been fully elucidated.

**Figure 1:**
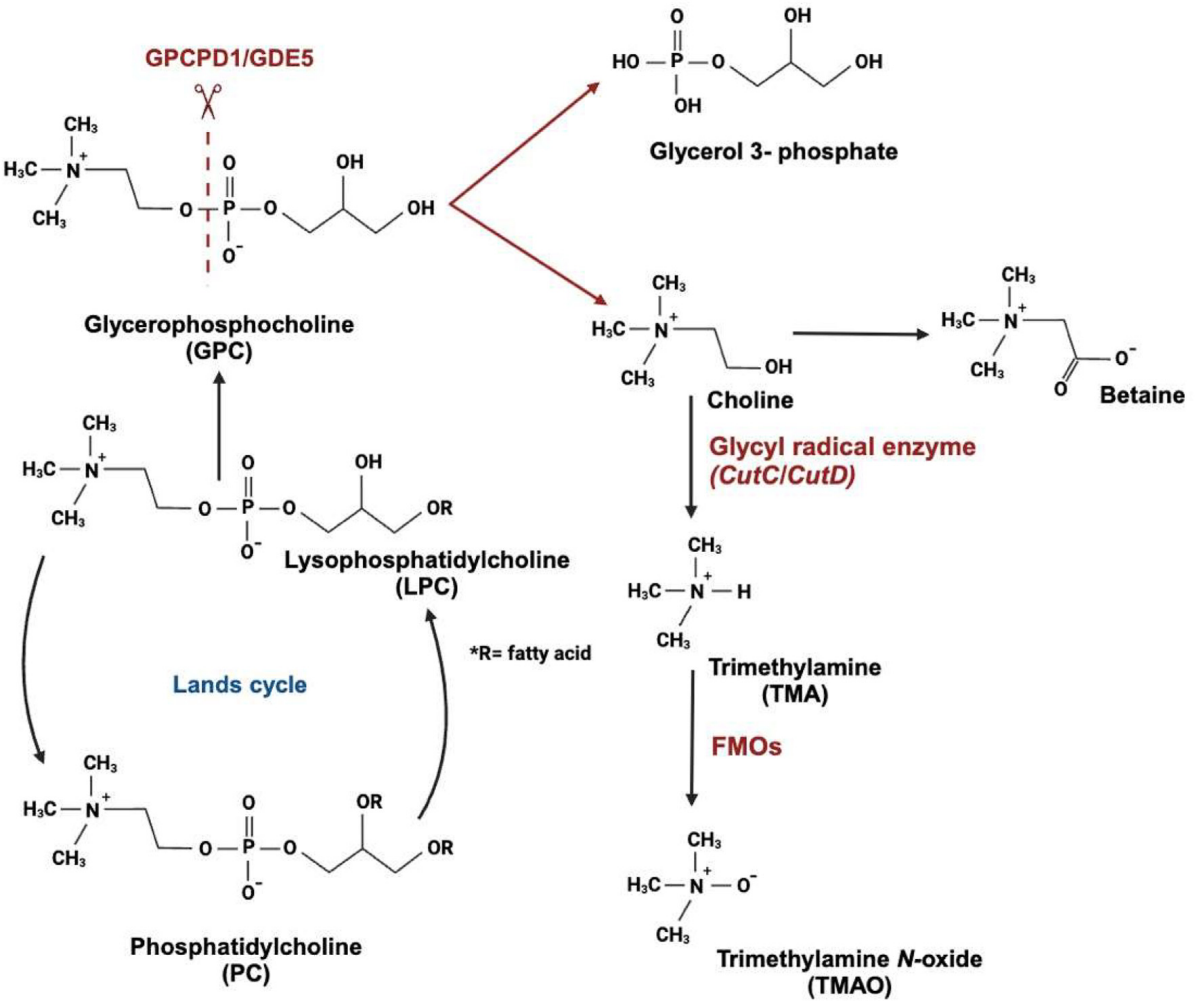
**GPC metabolism in mammalian cells.** GPC is an intracellular water-soluble metabolite of PC metabolism. In mammalian cells, GPC is hydrolyzed to choline and subsequently oxidized to betaine. In addition, choline can be metabolized to trimethylamine (TMA) by the intestinal microflora and subsequently converted to trimethylamine *N*-oxide (TMAO) in the liver. GPC, glycerophosphocholine; PC, phosphatidylcholine.

In this study, we examined TMAO production following GPC supplementation, and explored the mechanisms of GPC absorption and metabolism in the digestive tract *in vitro* and *in vivo*. Our data revealed that GPCPD1, which can hydrolyze GPC to choline, degrades GPC in conditioned medium from Caco-2 cells in the absence of gut microbiota, and that GPCPD1, originally identified as a cytosolic enzyme (9), can regulate extracellular choline metabolism. Here, we created intestine-specific Gpcpd1-deficient mice to elucidate its biological function in acute TMAO response, highlighting the host’s ability to modulate GPC metabolism and the potential decrease in the risk related to TMAO production.

## Results

### Intestinal metabolism and absorption of GPC *in vivo*

**Figure 2:**
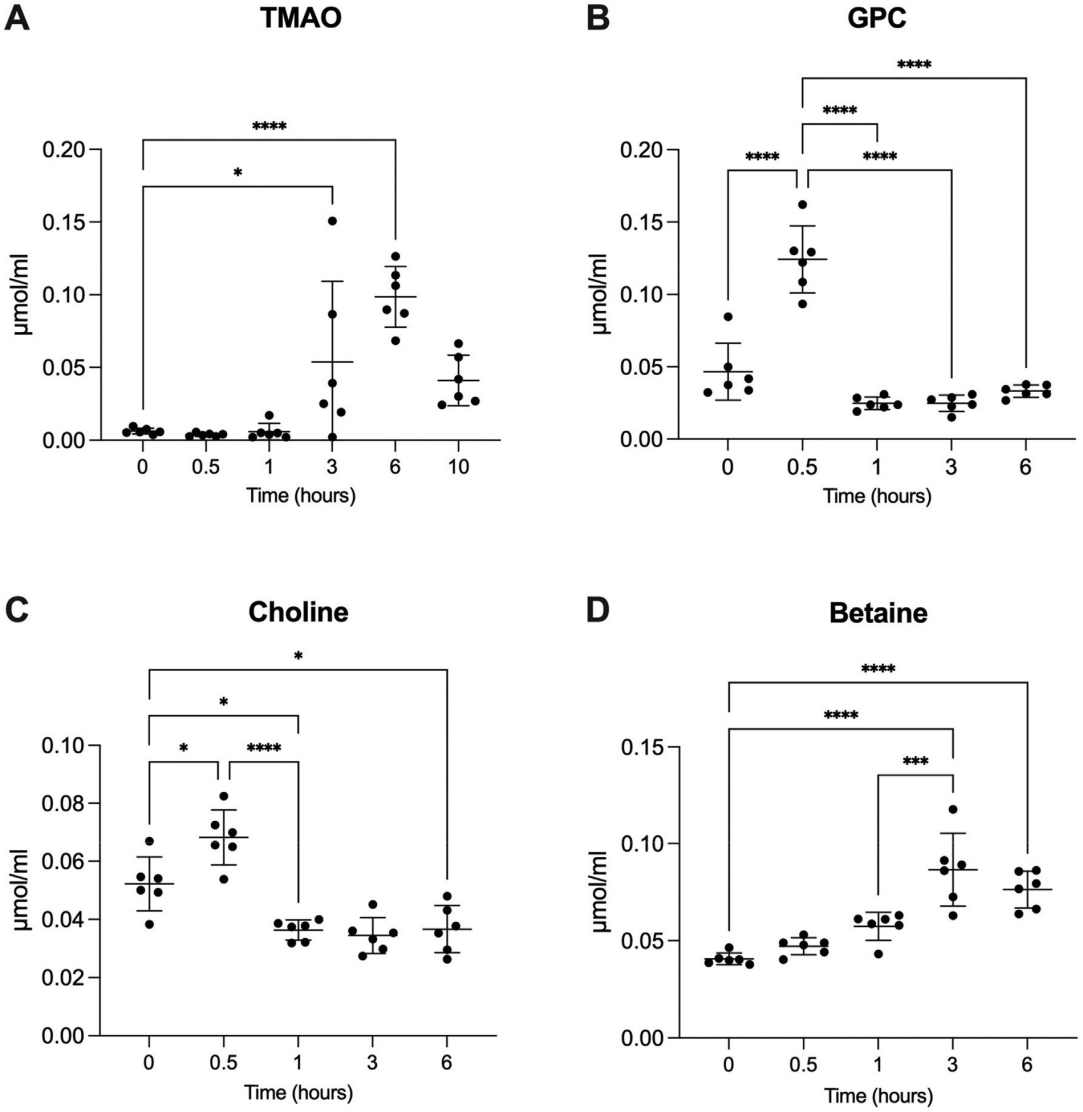
**Administration of glycerophosphocholine elevates circulating trimethylamine *N*-oxide level.***A*, circulating TMAO, (*B*) GPC, (*C*) choline, (*D*) betaine post oral GPC supplementation gavage (500 mg/kg body weight, n = 6). All values are presented as means ± SD; significance was determined using one-way ANOVA with Tukey’s multiple comparisons test. ∗*p* < 0.05, ∗∗*p* < 0.01, ∗∗∗*p* < 0.001, and ∗∗∗∗*p* < 0.0001.

To determine whether GPC supplementation contributes to increasing levels of TMAO in circulation, we administered GPC (500 mg/kg) to 6-week-old male mice (C57BL/6J) *via* oral gavage. Blood samples were collected from the caudal vein at intervals of 0.5, 1, 3, 6, and 10 h to quantify TMAO levels. We observed an increase in blood TMAO levels 3 and 6 h after gavage (Fig. 2*A*). Given the close association of GPC, choline, and betaine levels with TMAO levels, we also measured these choline metabolites in the blood. We noted a rapid increase in blood GPC and choline concentrations at 0.5 h postadministration (Fig. 2, *B* and *C*), whereas betaine concentrations increased at 3 and 6 h postadministration (Fig. 2*D*). These findings suggest that GPC can be readily metabolized to choline and partially but directly absorbed, thereby contributing to TMAO formation.

**Figure 3:**
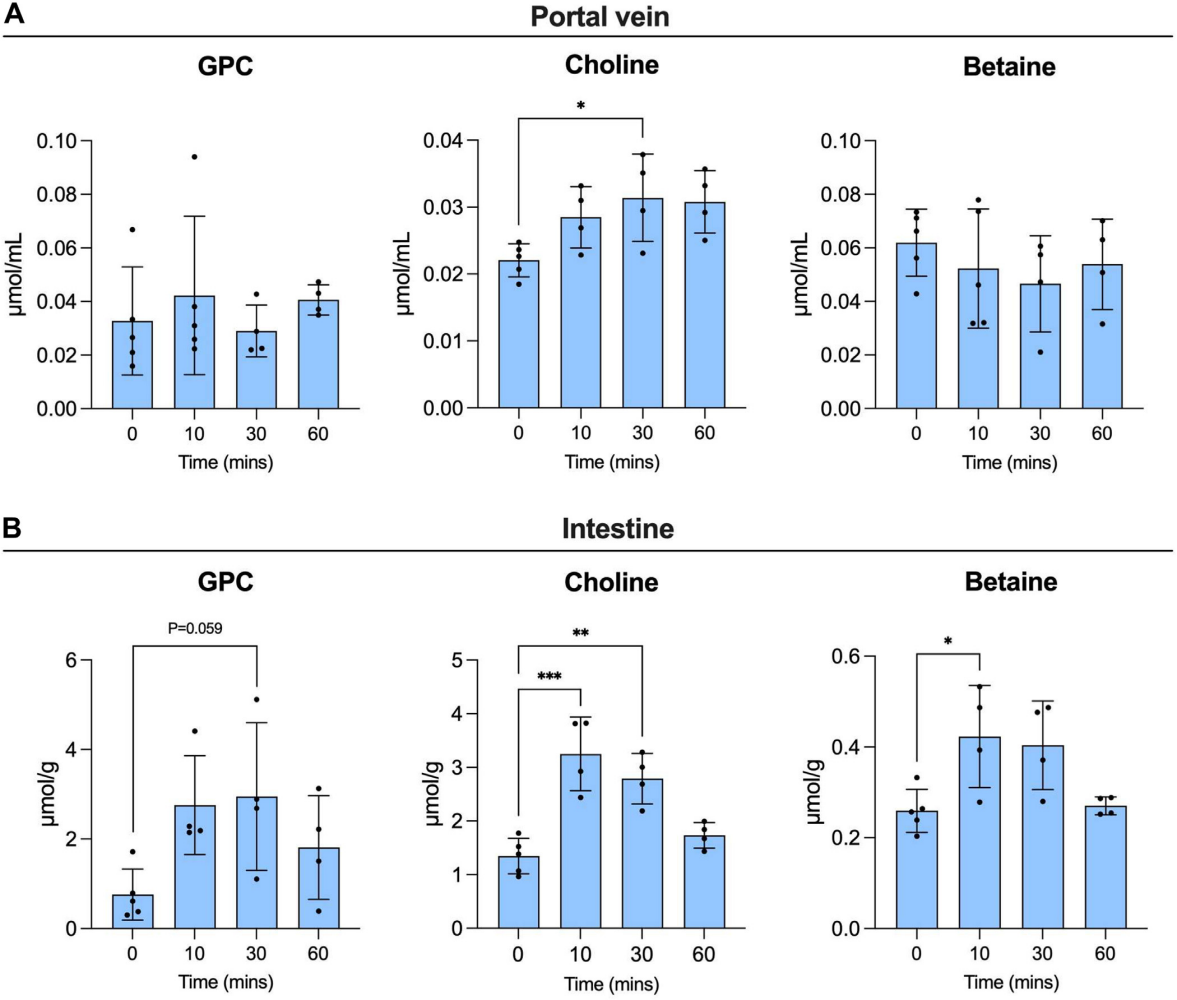
**GPC metabolism within the intestinal tract after intraduodenal GPC injection.** Intraduodenal injections of GPC (500 mg/kg body weight) changing choline metabolites in the (*A*) portal vein and in the (*B*) small intestine segment (n = 4–5). All values are presented as means ± SD; significance was determined using one-way ANOVA with Tukey’s multiple comparisons test. ∗*p* < 0.05, ∗∗*p* < 0.01, and ∗∗∗*p* < 0.001. GPC, glycerophosphocholine.

Because GPC is considered to be absorbed in the duodenum (23), we investigated the mechanism of GPC absorption in the intestinal tract *in vivo* by intraduodenal injections of GPC to mice. At 10, 30, and 60 min postinjection, we measured the GPC, choline, and betaine levels in the small intestine and portal vein. We observed no change in GPC levels in the portal vein (Fig. 3*A*), suggesting GPC hydrolysis in the intestinal tract. In contrast, there was a subsequent increase in choline levels in the portal vein and small intestine (Fig. 3, *A* and *B*). In addition, together with the data shown in Figs. 2 and 3, the elevated circulating GPC observed at 30 min postinjection is likely attributable to choline metabolism in other tissues, particularly in the liver.

### Extracellular hydrolysis involved in choline absorption from exogenously added GPC

**Figure 4:**
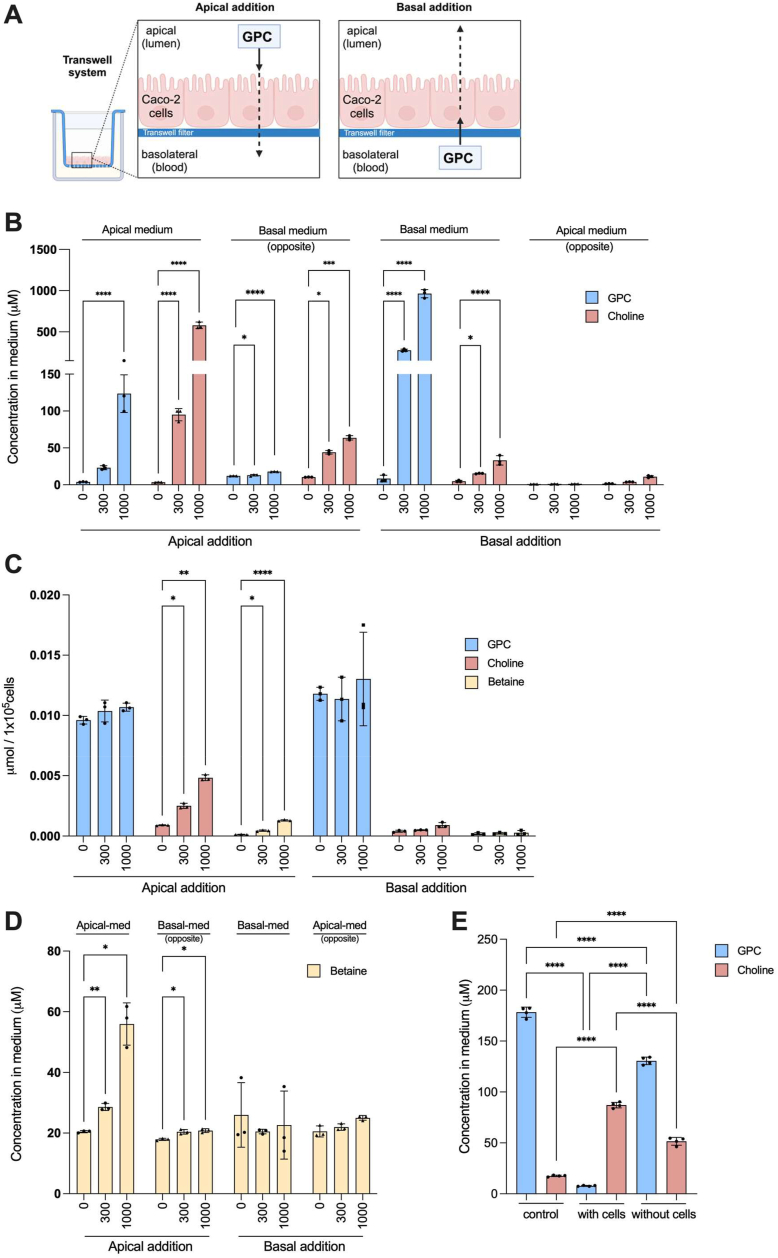
**Extracellular GPC hydrolysis for choline absorption in a Caco-2 cell monolayer system.***A*, schematic illustration of transwell system and experiment design. Choline metabolite levels in the (*B*) transwell system or in the (*C*) Caco-2 cells after apical addition or basal addition of GPC (100 μM, 300 μM, and 1 mM). *D*, betaine level in the transwell system after apical addition or basal addition of GPC. *E*, GPC-hydrolyzing activity in conditioned medium obtained from Caco-2 cells after 20 h of culture (without Caco-2 cells), conditioned medium with Caco-2 cells, and control medium. All values are presented as means ± SD (*A*) and (*E*), one-way ANOVA with Tukey’s multiple comparisons test. *B–D*, two-way ANOVA with Tukey’s multiple comparison test. ∗*p* < 0.05, ∗∗*p* < 0.01, and ∗∗∗*p* < 0.001, ∗∗∗∗*p* < 0.0001. GPC, glycerophosphocholine.

To investigate the mechanism of GPC absorption, we used human intestinal epithelial Caco-2 cells cultured in a Transwell system (Fig. 4*A*). GPC was added to the apical medium to assess its uptake by the epithelium. After 20 h, the apical medium, basolateral medium, and cell lysate were collected to quantify the choline metabolite levels. Intriguingly, we observed a significant increase in choline concentration in the apical space following the addition of 300 μM and 1 mM of GPC to the apical side of the Caco-2 cell monolayers, reaching concentrations of 95 μM and 579 μM, respectively, accompanied by a notable decrease in GPC level (Fig. 4*B*), which is possibly related to increase in betaine level in the apical space (Fig. 4*D*). Moreover, choline concentrations in the basolateral medium and cell lysate also increased (Fig. 4, *B* and *C*), suggesting that GPC may be hydrolyzed to free choline in apical medium. To confirm the origin of the increased free choline in the apical medium, we performed Caco-2 cell monolayer experiments with d9-GPC. At 4 h and 20 h after the addition of d9-GPC (50 μM) to the apical side, d9-choline and d9-betaine levels increased in the apical medium, while the d9-choline concentration increased in the basolateral medium (Fig. S1). To assess whether concentration-dependent passive diffusion through intracellular pathway is involved in the GPC transportation, GPC (300 μM and 1 mM) was added to the basolateral side of the Caco-2 cell monolayers. However, GPC concentration remained quite high on the basolateral side after 20 h of GPC addition, whereas choline concentration showed a slight increase (15.3 μM and 33.1 μM, respectively) (Fig. 4*B*). These suggest that GPC hydrolyzing activity on the basolateral side is limited and substantially lower than that on the apical side. Notably, neither the apical nor basolateral addition of GPC affected the intracellular GPC concentration of the Caco2 cells (Fig. 4*C*). Collectively, these results suggest that GPC does not pass through the membranes of intestinal epithelial cells through concentration-dependent diffusion. Because GPC is widely recognized to play an important role in regulating cellular osmotic pressure (24), it should not be readily transported and influence the osmotic potential as an organic osmolyte. However, further evidence is required to confirm this GPC transport mechanism in Caco-2 cell monolayers. This study indicated that GPC hydrolysis primarily occurs in the apical medium of Caco-2 cell monolayers, suggesting the physiological importance of luminal GPC metabolism.

To further characterize the extracellular GPC-hydrolyzing activity, we incubated GPC with conditioned medium obtained from the apical and basolateral sides of Caco-2 cells. Choline concentration increased only in the apical-conditioned medium (Fig. 4*E*), indicating the presence of enzymatic activity to hydrolyze GPC to choline on the lumen side of the intestine. Additionally, the GPC-hydrolyzing activity in the conditioned medium was lower than that in the presence of Caco-2 cells (Fig. 4*E*), suggesting that the GPC-hydrolyzing enzymes are localized not only in the extracellular space but also on the apical membrane surface of Caco-2 cells and regulate the extracellular choline concentration.

### GPCPD1 secreted from Caco-2 cells hydrolyzes GPC extracellularly

**Figure 5:**
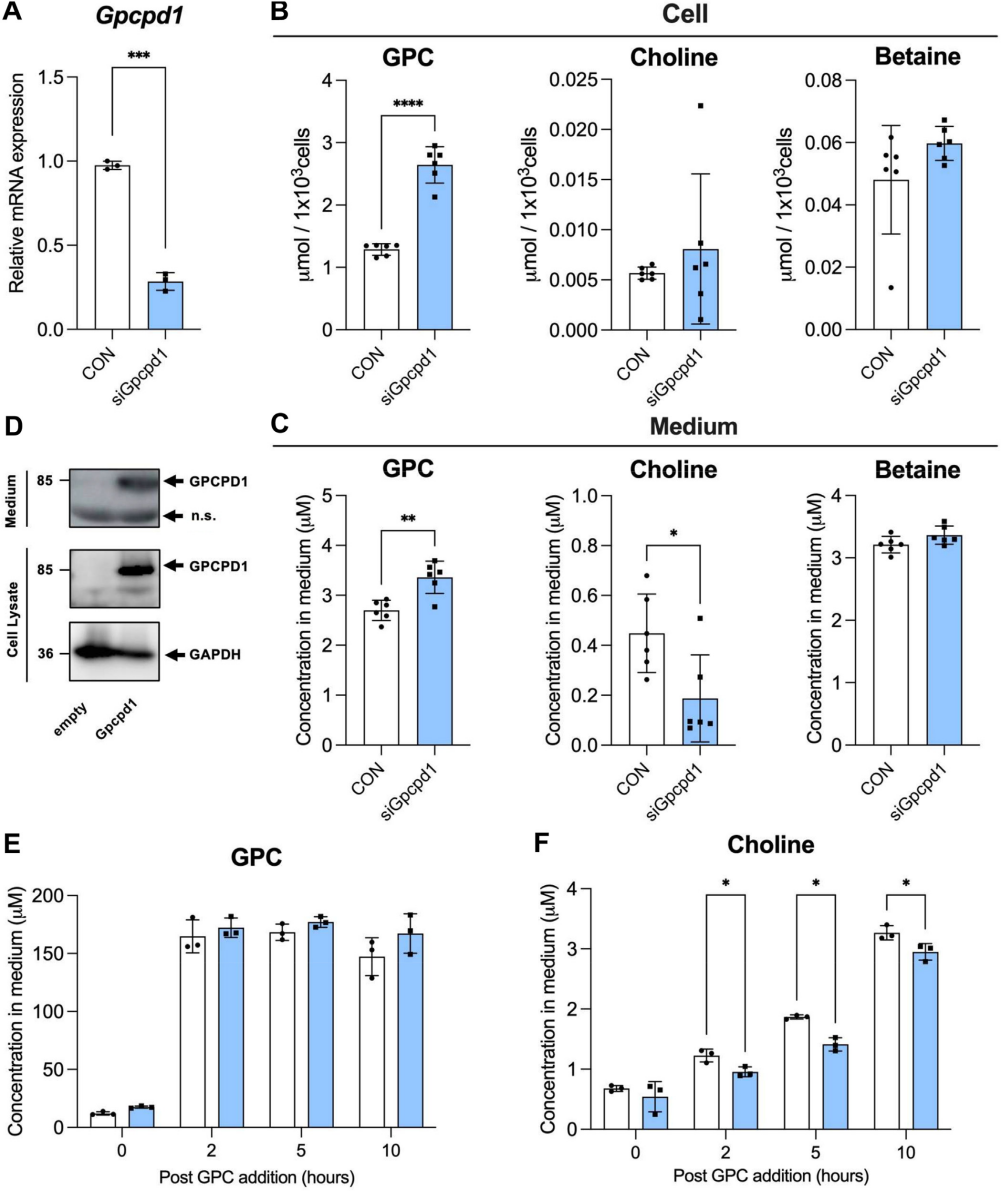
**Gpcpd1 knockdown in Caco-2 cells affects its GPC-hydrolyzing activity.***A-C*, Caco-2 cells were transfected with a negative control siRNA (CON) or *Gpcpd1* siRNA (siGpcpd1) (n = 4–5). *A*, *Gpcpd1* mRNA level in Caco-2 cells determined using quantitative PCR. Choline metabolite changes in the (*B*) Caco-2 cells lysate (n = 6) and (*C*) medium (n = 6) were quantified using LC-MS. *D*, Western blot analysis of medium and cell lysate from Caco-2 cells either transiently transfected with an empty vector or *Gpcpd1* expression vector. *E* and *F*, choline metabolite level after GPC addition (200 μM) to the medium from control Caco-2 cells or siGpcpd1-transfected Caco-2 cells quantified using LC-MS. All values are presented as means ± SD, (*A*), (*B*), and (*C*), unpaired two-tailed Student’s *t* test. *E* and *F*, two-way ANOVA with Tukey’s multiple comparison test. ∗*p* < 0.05, ∗∗*p* < 0.01, ∗∗∗*p* < 0.001, and ∗∗∗∗*p* < 0.0001. GPC, glycerophosphocholine; Gpcpd1, glycerophosphodiesterase 1.

Mammalian GDEs have recently been shown to be involved in multiple cellular signaling pathways by hydrolyzing glycerophosphodiesters, such as GPC and glycerophosphoinositol. We have previously identified GPCPD1, which selectively hydrolyzes GPC to choline and is widely expressed in mammalian tissues (Fig. 1) (9, 10). In this study, we used siRNA-mediated *Gpcpd1* knockdown in Caco-2 cells to examine its role in the GPC-hydrolyzing activity of these cells. We found that *Gpcpd1* siRNA transfection led to a reduction in both *Gpcpd1* mRNA and protein levels in Caco-2 cells (Figs. 5*A* and S2). *Gpcpd1* RNAi gene silencing resulted in significant GPC accumulation in cells and medium, as well as a significant decrease in choline levels in the medium (Fig. 5, *B* and *C*). These data indicate that Gpcpd1 can regulate choline homeostasis intracellularly, and that choline in the medium may compensate for the decreased choline production caused by GPCPD1 knockdown. Moreover, the presence of GPCPD1 protein in the culture medium suggests its potential role in regulating extracellular GPC levels (Fig. 5*D*). The GPCPD1 protein concentration in the extracellular space was relatively low compared with that within the cells; however, d9-GPC was hydrolyzed to d9-choline in the apical medium, as shown in Fig. S1. To further verify the involvement of GPCPD1 in GPC degradation in the Caco-2 cell culture medium, we added GPC (100 μM) to the conditioned medium from Caco-2 cells following siRNA transfection and measured the choline levels. As shown in Figure 5, *E* and *F*, the choline-producing capacity in the medium was reduced by *Gpcpd1* mRNA knockdown. Taken together, these observations suggest that GPCPD1 may play a direct role in regulating extracellular GPC levels.

### Targeted deletion of GPCPD1 in gastrointestinal epithelial cells alleviates circulating TMAO level in response to oral GPC administration

**Figure 6:**
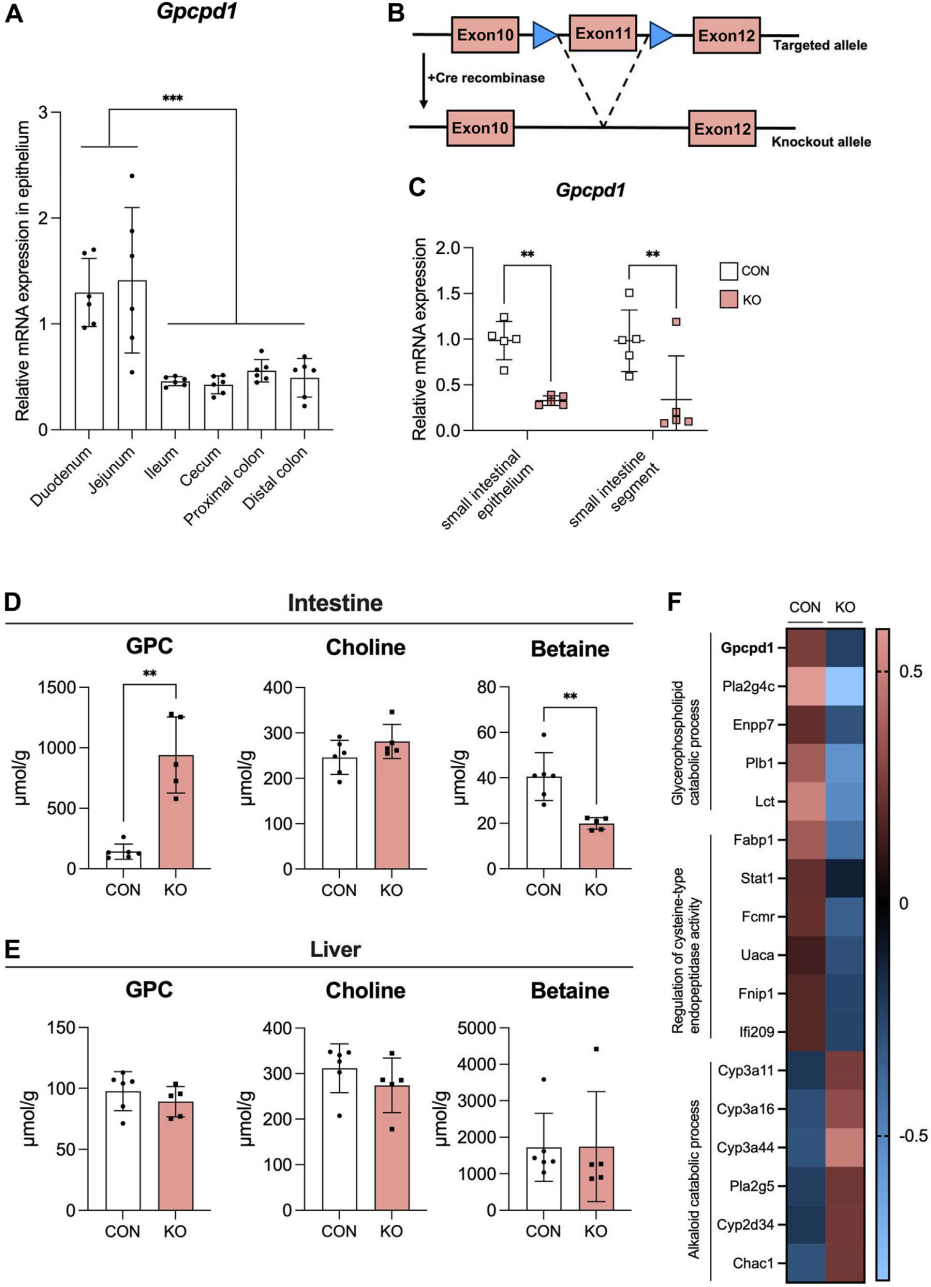
**Gpcpd1-KO mice show disrupted choline metabolism in the small intestine.***A*, epithelium from gastrointestinal segments (n = 6) was isolated and subjected to *Gpcpd1* mRNA analysis using qPCR. *B*, schematic illustration of Gpcpd1^flox/flox^, villin-cre (Gpcpd1-KO) mice. *C*, *Gpcpd1* mRNA levels of Gpcpd1-KO mice (KO) or its WT littermate (CON). Choline metabolite levels in the (*D*) intestine segment and (*E*) liver from Gpcpd1-KO mice (KO, n = 5) or its WT littermate (CON, n = 6) quantified using LC-MS. *F*, RNA-seq analysis of small intestine tissue from Gpcpd1-KO mice (KO) and its WT littermate (CON). Heat maps of genes associated with glycerophospholipid catabolic process and alkaloid catabolic process are shown. All values are presented as means ± SD, (*A*), one-way ANOVA with Tukey’s multiple comparisons test. *B*, two-way ANOVA with Tukey’s multiple comparison test. *D* and *E*, unpaired two-tailed Student’s *t* test ∗*p* < 0.05, ∗∗*p* < 0.01, ∗∗∗*p* < 0.001, and ∗∗∗∗*p* < 0.0001. Gpcpd1, glycerophosphodiesterase 1.

These results strongly suggested that Gpcpd1 plays a key role in GPC metabolism as a GPC-degrading enzyme in the intestinal tract. Quantitative PCR (qPCR) analyses were performed using total RNA derived from the epithelial layer of the gastric tract and showed that *Gpcpd1* mRNA is expressed in the small intestine (Fig. 6*A*). To create mice lacking *Gpcpd1* in an intestinal epithelial cell–specific manner (*Gpcpd1-*KO mice), *villin*-Cre mice expressing Cre protein specifically in intestinal epithelial cells were mated with GDE5*fl/fl* mice carrying *loxP* sequences at both ends of exon 11, which encodes the active center of the *Gpcpd1* gene (Fig. 6*B*). We isolated total RNA from the small intestine tissues of *Gpcpd1-*KO mice and analyzed *Gpcpd1* mRNA expression using qPCR (Fig. 6*C*). The results showed that Gpcpd1mRNA expression was significantly decreased in both the small intestine and the small intestinal epithelial cell layer of *Gpcpd1-*KO mice compared with that in WT mice.

**Figure 7:**
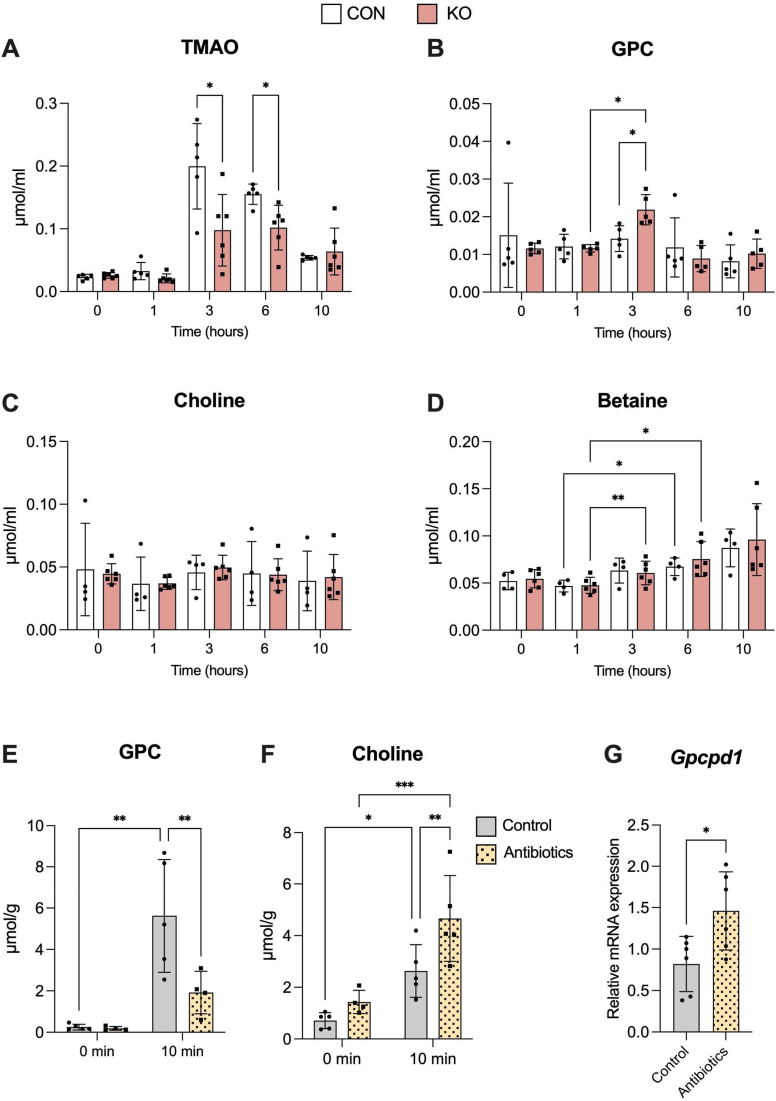
**Decreased circulating TMAO levels in Gpcpd1-KO mice.***A*, circulating TMAO, (*B*) GPC, (*C*) choline, and (*D*) betaine levels post oral GPC supplementation gavage (500 mg/kg body weight) in Gpcpd1-KO mice (KO, n = 6) or its WT littermate (CON, n = 4). *E* and *F*, changes in choline metabolite levels in response to intraduodenal injections of GPC (500 mg/kg body weight) with or without antibiotics treatment. *G*, intestinal *Gpcpd1* mRNA expression level with or without antibiotics treatment. All values are means ± SD, (*A*), two-way ANOVA with Tukey’s multiple comparison test. *B–F*, mixed-effects analysis with Šídák's multiple comparison test. *G*, unpaired two-tailed Student’s *t* test ∗*p* < 0.05, ∗∗*p* < 0.01, ∗∗∗*p* < 0.001, and ∗∗∗∗*p* < 0.0001. GPC, glycerophosphocholine; Gpcpd1, glycerophosphodiesterase 1; TMAO, trimethylamine N-oxide.

Next, choline metabolite levels in the small intestine and liver of *Gpcpd1*-KO mice were quantified using LCMS. As shown in Figure 6, *D* and *E*, in the small intestine, the GPC level was increased. Choline level was increased to a lesser extent in the small intestine of *Gpcpd1-*KO mice than in WT, whereas betaine levels decreased in Gpcpd1-KO mice. Previous reports have shown that betaine levels are an indicator of choline deficiency because the consumption of a choline-deficient diet affects betaine levels in tissues without any change in choline levels (25). In addition, there were no changes in hepatic choline metabolite levels, suggesting that the disrupted choline balance was limited to the small intestine of *Gpcpd1*-KO mice. In addition, DNA microarray analysis of RNA from intestinal tissues showed that the mRNA expression of genes related to glycerophospholipid metabolism was affected by *Gpcpd1* deficiency (Fig. 6*F*). Since *Pla2g* and *Plb1* expression are reportedly involved in the deacylation process during phospholipid remodeling (Lands’ cycle), which affects the diversity of PC fatty acid composition (26), we measured the levels of linoleic acid–rich PC (16:0–18:2) and docosahexaenoic acid-rich PC (16:0–22:6) in the gut of *Gpcpd1*-KO mice. Although no differences were observed in these levels between *Gpcpd1*-KO mice and WT mice (CON), as shown in Fig. S3, further research is required to better understand PC metabolism through lipidomic analyses. To examine its potential role in regulating circulating TMAO production, GPC (500 mg/kg) was orally administered to *Gpcpd1*-KO and wild-type mice, and caudal blood was collected for LC-MS analysis. Although no change in blood choline concentration was observed, blood TMAO concentration was significantly lower in *Gpcpd1*-KO mice than in than mice, suggesting that Gpcpd1 expressed in intestinal epithelial cells degrades GPC to choline and is involved in TMAO production (Fig. 7*A*). Interestingly, blood GPC levels in *Gpcpd1*-KO mice increased 3 h after GPC supplementation, suggesting an indirect effect of gut GPCPD1 activity on circulating GPC levels (Fig. 7, *B–D*).

Finally, to address the involvement of intestinal bacteria in the degradation of GPC to choline, GPC was administered to the duodenum of mice that had received a mixture of four antibiotics (ampicillin, neomycin, metronidazole, and vancomycin) for 3 weeks. Since there was a significant increase in choline levels in the small intestine 10 min after GPC injection (Fig. 3*B*), we measured choline metabolite levels in the small intestinal tissue at the same time point (Fig. 7, *E* and *F*). Antibiotic treatment decreased the GPC concentration in small intestinal tissue, which was accompanied by an increase in choline levels (Fig. 7, *E* and *F*). These results suggest that intestinal bacteria are not directly involved in the degradation of GPC to choline and that the GPC-hydrolyzing activity in intestinal tissue is upregulated by antibiotic treatment. Interestingly, this study also showed that intestinal *Gpcpd1* mRNA expression was significantly increased following antibiotic treatment (Fig. 7*G*). These observations indicate that gut bacteria may regulate GPC-hydrolyzing activity, possibly *via* GPCPD1, in small intestinal tissues. In contrast, antibiotic treatment abolished the increase in intestinal TMA production in response to duodenal GPC administration (Fig. S4), which supports the role of gut bacteria in the conversion of choline to TMA.

## Discussion

Choline is widely recognized as an essential nutrient for various physiological functions. According to the National Academy of Medicine, the daily adequate intake of choline for healthy adult men is set at 500 mg, whereas the European Food Safety Authority recommends a slightly lower intake of 400 mg (27, 28). However, recent analyses have shown that a large proportion of the population, including pregnant and lactating women, fails to meet these recommendations, highlighting the importance of choline supplementation (6). In recent reports, it has been widely proposed that GPC, with its water solubility, crosses the blood–brain barrier and improves brain activities, such as memory function, in animal experiments and human clinical studies (29, 30). Although, based on our understanding, cognitive benefits are supposedly associated with increased acetylcholine levels in the hippocampus, the exact mechanism of GPC absorption and metabolism in the digestive ducts is not understood. In this study, experiments using Caco-2 cells showed a slight but significant increase in GPC concentration in the basolateral medium when GPC was added to the apical medium without any change in the intracellular GPC levels. In addition, the GPC levels in the intestinal segment tended to increase in response to GPC supplementation. Taken together, these results suggested that GPC was partially transferred across the epithelium by passing through the intercellular spaces between Caco2 cells.

GPC is widely recognized not only as an important intermediate in the PC degradation pathway but also as a major intracellular form of stored choline. Recent studies have revealed that GPC plays a key role in modulating phospholipid membrane metabolism and turnover. In yeast, phospholipase B-mediated hydrolysis of PC produces GPC, which is then reacylated by GPC acyltransferase and lysophosphatidylcholine acyltransferase to form PC, resulting in changes to the composition of monounsaturated and diunsaturated PC species (31, 32). In mammals, abnormal choline metabolism, particularly alterations in GPC levels, has been actively studied in relation to carcinogenesis and tumor progression. Recent studies have focused on the roles of GPC-degrading enzymes to better understand the biological importance of intracellular GPC metabolism in the proliferation and migration of cancer cells *in vitro* (33, 34). Interestingly, this study demonstrates that *Gpcpd1*, an endogenous GPC-degrading enzyme, modulates GPC metabolism extracellularly, independent of the intestinal microbiome, underscoring the importance of the luminal environment as the primary site for GPC metabolism. We have previously identified Gpcpd1/GDE5 as a cytosolic GDE that selectively hydrolyzes GPC (9). The Gpcpd1 protein contains a catalytic domain that shares a common motif (amino acids 320–385) with other GDEs; however, it lacks a transmembrane region typical of other mammalian GDEs. Interestingly, it has an N-terminal carbohydrate-binding domain that is often found in glycosylhydrolases, such as amylases, indicating the possibility that Gpcpd1 can be secreted into the intestinal lumen and function as a digestive enzyme in GPC hydrolysis. Furthermore, GPC-hydrolyzing activity was significantly higher in the culture medium in the presence of Caco-2 cells than in the conditioned medium alone (Fig. 4*E*), suggesting that enzymes localized on the membrane surface of Caco-2 cells are also responsible for the hydrolysis of GPC to choline. Among the six GDE family genes expressed in Caco-2 cells, GDE2 (also known as GDPD5) is particularly notable for its localization to intracellular vesicles and the plasma membrane (35, 36, 37). Interestingly, previous studies on renal tubule epithelial cells have shown the role of GDE2 in regulating intracellular GPC levels in response to hyperosmolarity induced by high extracellular urea concentrations (35). In the context of intestinal epithelial cells, because *GDE2* mRNA is actually expressed in Caco2 cells, GDE2-derived enzymatic activity might be involved in the degradation of GPC to choline on the surface membrane of Caco-2 cells.

Clinical reports using stable isotope-labeled GPC showed that orally administered GPC increased blood choline concentration 1 h after GPC administration, followed by an increase in the blood concentration of TMAO 5 to 9 h later (38). In the context of circulating TMAO levels, the role of the bacterial composition and abundance of microorganisms in the gastrointestinal tract should also be discussed for GPC metabolism because the composition and abundance of microorganisms in the gastrointestinal tract are widely recognized to play a crucial role in regulating nutrient digestion, absorption, and stability in the digestive tract. Previous studies have indicated that gut microbes, particularly those harboring CutC/D bacteria, are involved in GPC metabolism to TMA because of their TMA moiety metabolism (39). However, in comparison with GPC, choline can be more efficiently converted to TMA in the cecum (23). On the other hands, it is well reported that bacterial GlpQ and UgpQ play vital roles in hydrolyzing glycerophosphodiesters with broad substrate specificities, such as GPC and glycerophosphoinositol, to glycerol 3-phosphate, which is an important carbon and phosphate source for bacteria (40). Incubation of intestinal bacteria, such as *Escherichia coli*, *Proteus mirabilis*, and *Lactobacillus acidophilus,* with GPC yielded choline and glycerol 3-phosphate (23). However, UgpQ in *E. coli* is upregulated in response to phosphate deprivation and GlpQ expression in *Bacillus subtilis* increases under phosphate-deficient conditions (40, 41, 42). Given the constant abundance of phosphate in the intestinal tract, GlpQ and UgpQ expression in intestinal bacteria may be limited, suggesting that Gpcpds from intestinal epithelial cells primarily degrade GPC to choline. A previous report by Inaba *et al.* showed that *in vivo* rat intestinal perfusion with GPC resulted in a significant increase in choline levels in the intestinal perfusate (43). Taken together, although the results of our experiment involving the use of antibiotics suggest that gastrointestinal host cells play an important role in GPC metabolism, further analysis is needed to directly assess the involvement of the Gpcpd1 enzymatic activity of intestinal epithelial cells in GPC hydrolysis in the intestinal lumen.

Sufficient choline supply is exceptionally important for preterm infants (44). Choline is actively transported *via* the placenta to the fetus during pregnancy (45). In preterm infants, the composition of the gut microbiota undergoes rapid changes during infancy and eventually stabilizes upon the introduction of solid foods (46, 47). Water-soluble forms of choline, such as GPC, primarily from breast milk, serve as an important source of choline for infant development (48). GPC can be absorbed and metabolized in the neonatal digestive system independent of the influence of the gut microbiome. More importantly, this study proposes the existence of basic regulatory mechanisms within the digestive tract involving host enzymes that facilitate the efficient absorption and metabolism of GPC for fetal and infant development during early infancy.

## Experimental procedures

### Animal experiments

All animal experiments were conducted in accordance with the animal care protocol approved by the Animal Use Committee of the Hiroshima University (Ethical approval No. C22-30). All animals were housed at 24 to 26 °C in a 12 h light-dark cycle (8:00-20.00 light cycle, 20.00-8.00 dark cycle) and fed with commercial chow diet (MF, Oriental Yeast Co, Ltd) and water *ad libitum*. For GPC supplementation experiments, male C57BL/6J mice aged 8-week-old (∼24 g) were obtained from Charles River. We previously created Gpcpd1 *flox/flox* mice in the C57BL/6 background using CRISPR–Cas9 and PITCh (Precise Integration into Target Chromosome) system with microhomology-mediated end joining–directed plasmid by flanking exon 11 with loxP sites (10, 49). To generate an intestinal epithelial-specific Gpcpd1-deficient mouse, Gpcpd1 *flox/flox* mice were crossed with villin-Cre mice in C57BL/6 background. Male villin-Cre; Gpcpd1 *flox/flox* mice with ages of 15- to 18-week-old were used in this study with Gpcpd1 *flox/flox* littermates as controls. The primers used for genotyping were as follows: villin-Cre, F, 5′-CAAGCCTGGCTCGACGGCC-3′, and R, 5′-CGCGAACATCTTCACGTTCT-3′; Gpcpd1 flox, F, 5′-GGGCATTCAATCCCATTACAG-3′; and R, 5′-CCTCCAAATGACTTCATACTGGC-3′. GPC was orally administrated to male mice using 1.5-inch 20 gauge feeding needle (500 mg/kg body weight). The blood was collected from caudal veins 0, 1, 3, 6, and 10 h after the GPC administration. The GPC dosage was estimated to be approximately equal to the daily intake of choline bitartrate in the AIN93 diet.

### Duodenal GPC injection in mice

GPC solution was injected directly into the duodenum of the male C57BL/6J mice (500 mg/kg body weight) under isoflurane anesthesia. Blood was collected from portal veins 0, 10, 30, and 60 min after the injection. Intestine segment was collected, washed with saline, and stocked in liquid nitrogen immediately for later quantification of the choline metabolites.

### Antibiotics treatment in mice

Eight-week-old male C57BL/6J mice were randomly divided into two groups: water group and antibiotics group (n = 5). The antibiotics group was administrated with an antibiotic cocktail (ampicillin 1 mg/l, neomycin 1 mg/l, metronidazole 1 mg/l, and vancomycin 0.5 mg/l) *via* drinking water for 21 days. After the antibiotics treatment, mice were subjected to duodenal GPC injection (500 mg/kg body weight) under isoflurane anesthesia. Blood was collected from portal veins 10 min after the injection.

### Caco-2 cell culture experiment

Caco-2 cells (HTB-37; American Type Culture Collection) were cultured in Dulbecco's modified Eagle's medium (DMEM) supplemented with 10% fetal calf serum, 100 units/ml penicillin, and 100 μg/ml streptomycin under a humidified atmosphere of 5% CO_2_ in air at 37 °C. Caco-2 cells were seeded into permeable polyester membrane filter supports (Corning transwell, 12-mm diameter, 0.4-μm pore size) at a density of 0.25 × 106 cells/cm^2^ and maintained for 14 days under a humidified atmosphere of air, 5% CO2, and at 37 °C. The cell culture medium was refreshed every 3 days. Before the experiments, Caco-2 cell monolayers grown in transwell inserts were gently rinsed with choline chloride–free DMEM supplemented with 10% fetal calf serum (final choline concentration: 4.4 μM). Fresh medium was replaced on the apical and basolateral sides of transwells before the experiment. Before starting the experiment, transepithelial electrical resistance voltage was measured to ensure membrane integrity (2234 ± 201 Ω-cm^2^). GPC was added to the apical or basolateral side of the chamber to start the transport experiments at GPC concentration of 100 μM, 300 μM, and 1 mM. Medium samples were taken from the apical and basolateral sides. Caco-2 cells were gently washed with choline chloride–free DMEM twice and homogenized using a mixture of water and methanol with a ratio of 1:4 (vol/vol) and transferred to a screw capped glass tube. The conditioned medium obtained by culturing Caco-2 cells and culture medium in the presence of Caco-2 cells were incubated with 200 μM GPC to examine the GPC-hydrolyzing activity. These samples were subjected to LC-MS analysis for the quantification of choline metabolites as described below. A complementary DNA encoding full-length mouse Gpcpd1/GDE5 was cloned into mammalian expression vector pEF/myc/cyto (Invitrogen), generating pEF-Gpcpd1. DNA transfections were performed with pEF-Gpcpd1 or pEF/myc/cyto as an empty vector using GeneJuice Transfection Reagent (Merck & Co, Inc) according to the manufacturer's instructions. Stealth siRNA duplex oligoribonucleotides against human Gpcpd1 were synthesized by Invitrogen. The sequences were as follows: sense, 5′-CAGUGUGUUGUGGAAAGCAGUGAUU-3′; antisense, 5′-AAUCACUGCUUUCCACAACACACUG-3′. Caco-2 cell were grown with choline chloride–free DMEM supplemented with 10% fetal calf serum for 4 h and transfected with these siRNAs to a final concentration of 20 nM using Lipofectamine RNAimax (Invitrogen).

### Isolation of gastrointestinal epithelial cells

Gastrointestinal epithelial cells were isolated from whole colonic tissues (50). Briefly, intestinal and colonic tissues (3 cm in length) were washed with saline and cut into 5 or 6 small segments and transferred into 3 ml isolation buffer (Ca^2+^- and Mg^2+^-free Hanks' balanced salts solution supplemented with 0.1 mmol DTT/L, 5 mmol EDTA/L, as well as protease and phosphatase inhibitors) and incubated for 1 h with gentle agitation and then vigorously shaken to release the epithelial cells. The supernatants were centrifuged at 5000*g* for 2 min at 4 °C to sediment the epithelial cells for RNA experiment.

### RNA extraction and qPCR analysis

Total RNA was extracted using QIAzol and purified with an RNeasy Lipid Tissue Mini Kit (Qiagen Sciences). Reverse transcription was conducted with ReverTra AceTM (TOYOBO), random primers (TOYOBO), and dNTPs (TOYOBO). For qPCR analysis, complementary DNA and primers were added to the THUNDERBIRD SYBR qPCR Mix (TOYOBO). PCs were then performed using StepOnePlus (Thermo Fisher Scientific Inc). The primers used for PCR analyses were as follows: mouse Gpcpd1, F, 5′- ATTGGACGTTGGACATCGTG-3′, and R, 5′-GAGGTCATGATACACCACGG-3′; human Gpcpd1, 5′-GCTTAAGAGCTGTTTGTGTC-3′, and R, 5′-CCCCAAAATGACCTCTGTGC-3′; L19, F, 5′-GGCATAGGGAAGAGGAAGG-3′, and R, 5′-GGATGTGCTCCATGAGGATGC-3′.

### DNA microarray analysis

Total RNA from the intestine of WT and Gpcpd1-KO mice at 20 weeks of age was isolated using RNeasy Lipid Tissue Kit and subjected to circular RNA synthesis. Fluorescence labeling, hybridization, and image processing were performed according to the manufacturer’s instructions (Agilent Technologies). Briefly, circular RNAs were fragmented and hybridized on the 44 K whole mouse genome oligo microarray slides at 65 °C for 17 h. The glass slides were then washed and scanned using Agilent DNA microarray scanner (Agilent Technologies) as previously described (9). Gene expression data were obtained by the Agilent Feature Extraction Program (version 9.5). To organize functionally related genes into biologically meaningful modules, we use Gene Ontology Enrichment Analysis for efficient interpretation. Twenty highest modules with fold enrichment >1.90 were selected.

### Western blot analysis

Cell lysate and culture medium from Caco-2 cells were used for Western blot analysis. Cell lysate was prepared with RIPA buffer containing 1 mM PMSF, 20 μg/ml aprotinin, and 10 μg/ml phosphatase inhibitor cocktail. The lysates were centrifuged at 13,000*g* for 15 min at 4 °C, and the supernatant was collected. Protein concentration was quantified using DC Protein Assay (Bio-Rad Laboratories, Inc). Fifteen micrograms of cell lysate and 10 μl of culture medium were boiled at 92 °C for 3 min. Equal amounts of protein were then loaded into the wells of the 10% SDS-PAGE gel and run for 100 min at 24 to 26 mA. Then, the protein was transferred onto a polyvinylidene difluoride membrane using semidry method at 2 mA x membrane area for 120 min. The membranes were next blocked with 4% skim milk in 1x PBS for 1 h, and then incubated in primary antibody overnight at 4 °C for anti-Gpcpd1 (1:1000, Okazaki *et al.* 2010) and anti-GAPDH (1:5000, WAKO). Can Get Signal Immunoreaction Enhancer Solution (TOYOBO) were used to incubate antibodies. Protein bands were detected using Western Lighting-ECL (PerkinElmer, Inc).

### LC-MS/MS quantitation of choline metabolites

The extraction method of Bligh and Dyer was used (51). Intestine tissues were homogenized in H_2_O, added with 2 ml of 2:1 methanol-CHCl_3,_ and vortexed for 15 s. Homogenates were then centrifuged at 2000 rpm for 10 min. The remaining pellets were added with 2.5 ml of 2:1:0.8 methanol-CHCl_3_-H_2_O mix, vortexed, and centrifuged at 2000 rpm for 10 min. The supernatants were mixed with previously obtained supernatants and added with CHCl_3_ and H_2_O, 1.3 ml of each. The supernatant mixtures were vortexed and separated into two layers after being centrifuged at 2000 rpm for 10 min. The water-soluble parts were evaporated at 60 °C for 6 h. Pellets were added with 250 μl MeOH, vortexed, and centrifuged at 10,000 rpm for 5 min Two hundred microliters supernatants were collected and mixed with 800 μl methanol and stored for measurement. Caco-2 cells were scraped manually using methanol and transferred to a screw-capped glass tube. Plasma and culture medium from Caco-2 cells were mixed with four volumes of methanol containing isotope labeled compounds, vortexed for 15 s, and then centrifuged at 2000 rpm for 10 min. The supernatants were collected and stored for measurement. For the quantitation of choline metabolites, mobile phase A composed of 95% CH_3_CN, 5% MiliQ water, 5 mM HCOONH_4_ (pH 3.0), and mobile phase B composed of 10% CH_3_CN, 90% MiliQ water, 5 mM HCOONH_4_ (pH 3.0) was prepared for measurement. Water-soluble samples were separated based on gradient generated from mobile phase A and B using Acquity UPLC BEH HILIC column (Waters). Ion quantification was performed using electrospray ionization method in positive mode with choline at 25 V cone voltage, *m/z* = 104, betaine at 25 V cone voltage, *m/z* = 118, and GPC at 20 V cone voltage, *m/z* = 258. The results were either adjusted to analyze tissue weights or the cell density. Peak area ratios of the analyte to the internal standard (d9-choline, d9-GPC, and d9-betaine) were calculated as a function of the concentration ratios of the analyte (QuanLynx, Waters). For the quantitation of TMAO and TMA, mobile phase A composed of 10 mM HCOONH_4_ in MiliQ water (pH 3.0) and mobile phase B composed of 100% acetonitrile were used for measurement. Samples were separated based on the gradient generated from mobile phase A and B using a reversed-phase BEH C18 column (Waters). Ion quantification was performed at 25 V cone voltage and 5V collision energy. Analyte detection was performed using multiple reaction monitoring in positive mode with the following transition: *m/z* 75.9 → 58.1 for TMAO and *m/z* 60.1 → 44.15 for TMA. The results were adjusted to dilution time. Peak area ratios of the analyte to the internal standard (d9-TMAO and d9-TMA) were calculated as a function of the concentration ratios of the analyte (QuanLynx, Waters).

### Statistical analysis

All data are expressed as the mean ± SD. Individual data points are shown in all graphs, with at least three independent biological replicates were used for each experiment. Statistical analyses were conducted by Student's *t* test and two-way ANOVA using GraphPad Prism 9 software (https://www.graphpad.com/), with *p* values < 0.05 are considered statistically significant as denoted in the figure legends.

## Data availability

All data are available from the corresponding authors upon reasonable request.

## Supporting information

This article contains supporting information.

## Conflict of interest

The authors declare that they have no conflicts of interest with the contents of this article.
