# Supplementary material for: Role of Gpcpd1 in intestinal alpha-glycerophosphocholine metabolism and trimethylamine N-oxide production
Source: J Biol Chem. 2024 Nov 5;300(12):107965. doi: 10.1016/j.jbc.2024.107965 (PMC11652876; doi:10.1016/j.jbc.2024.107965)
Supplement: Supplementary Figs. S1–S5 [file mmc1.docx]

**Role of Gpcpd1 in intestinal alpha-glycerophosphocholine metabolism and trimethylamine *N*-oxide production**

Siyi Chen, Shiho Inui, Rahmawati Aisyah, Ryoko Nakashima, Tatsuya Kawaguchi, Minori Hinomoto, Yoshiko Nakagawa, Tetsushi Sakuma, Yusuke Sotomaru, Noriyasu Ohshima, Thanutchaporn Kumrungsee, Takeshi Ohkubo, Takashi Yamamoto, Yutaka Miura, Takuya Suzuki, Noriyuki Yanaka*

*To whom correspondence should be addressed: Noriyuki Yanaka, Graduate School of Integrated Sciences for Life, Hiroshima University, Higashi-Hiroshima, 739-8528, Japan. Tel.: +81-82-4247979; Fax: +81-82-4247916; E-mail: yanaka@hiroshima-u.ac.jp

Supplementary Figures:

Supplementary Figure S1

Supplementary Figure S2

Supplementary Figure S3

Supplementary Figure S4


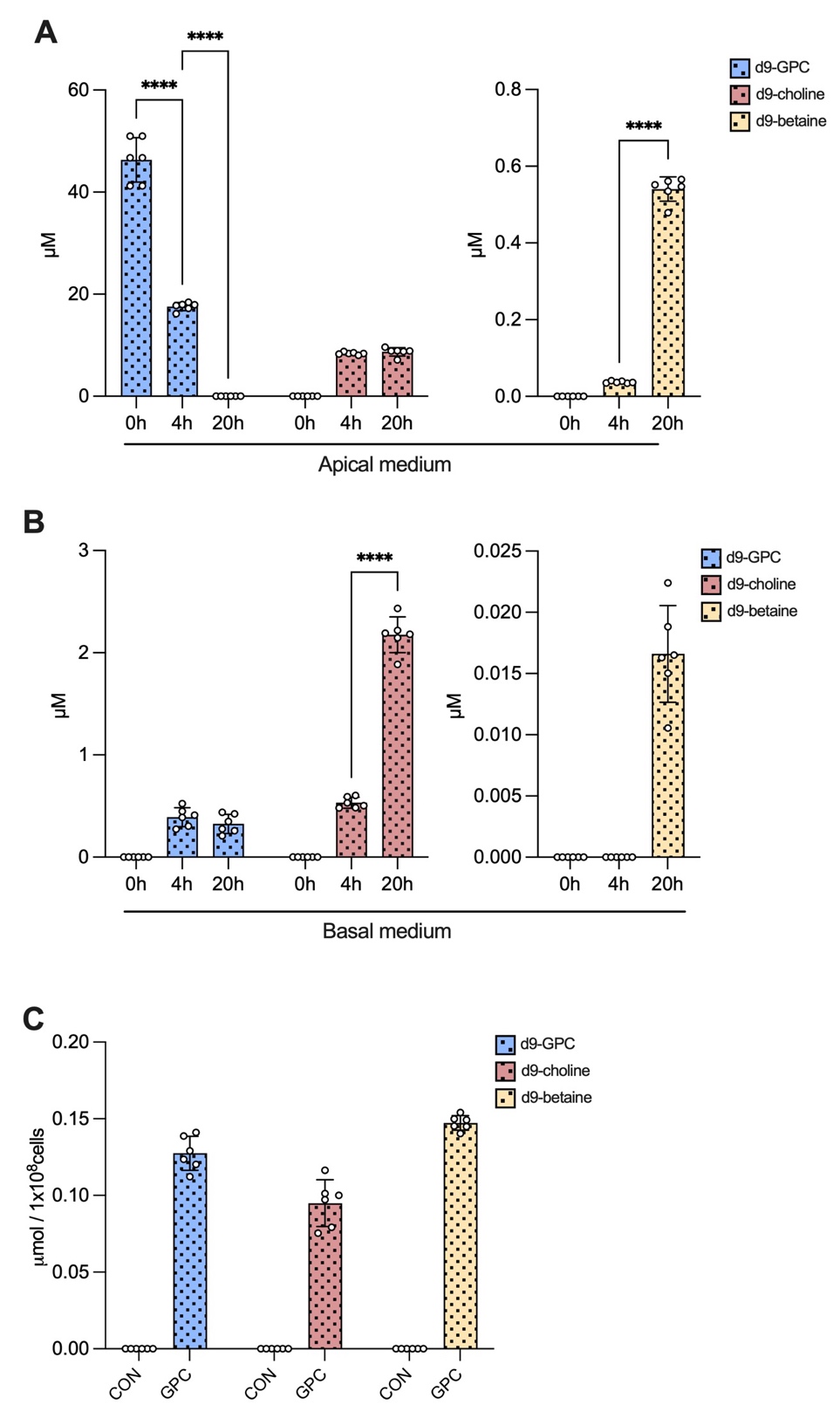


**Figure S1. GPC flux into choline and betaine.** d9-GPC, d9-choline, and d9-betaine levels in the **(A)** apical medium (n=6), **(B)** basal medium (n=6), and **(C)** Caco-2 cells (n=6) after apical d9-GPC (50uM) addition. All values are means ± SD**.** Statistical analysis was performed using two-way ANOVA with Tukey’s multiple comparison test. *p < 0.05, **p < 0.01, ***p < 0.001, ****p < 0.0001.


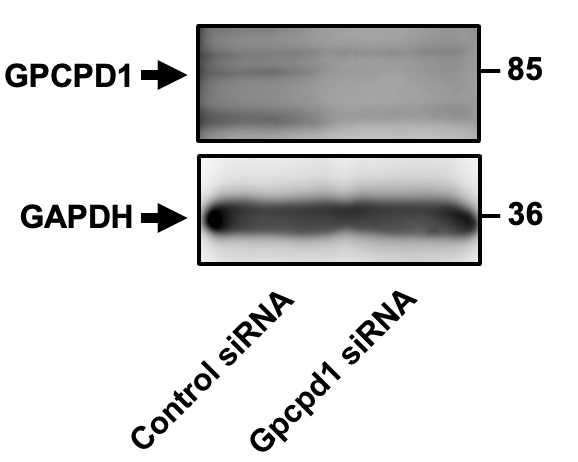


**Figure S2. GPCPD1 protein expression in Caco-2 cells.**


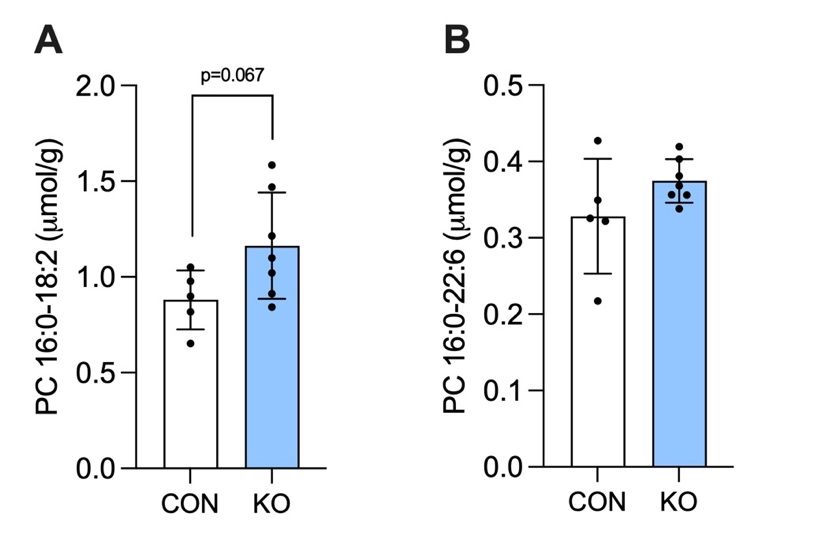


**Figure S3. Intestinal PC profile of Gpcpd1 KO mice.** Quantification of **(A)** PC 16:0-18:2, and **(B)** PC 16:0-22:6 in the gut of Gpcpd1 KO mice (KO, n=6) and its wild-type littermate (CON, n=5). All values are means ± SD. Statistical analysis was performed using Student’s t test.


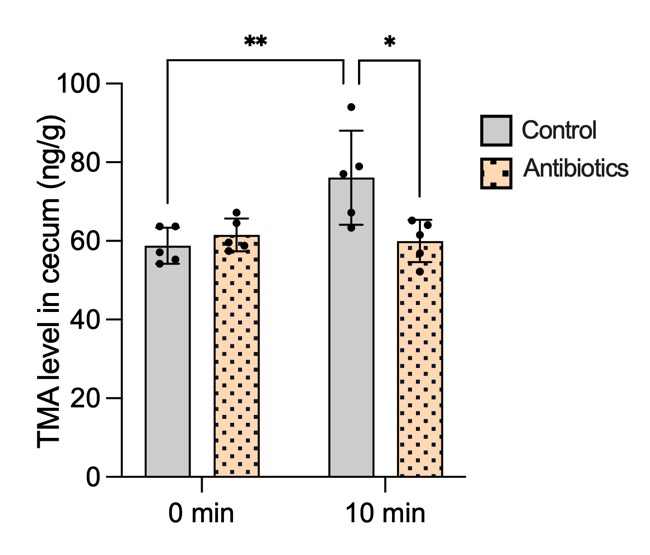


**Figure S4. TMA level in cecum after GPC administration.** All values are means ± SD. Statistical analysis was performed using two-way ANOVA with Tukey’s multiple comparison test. *p < 0.05, **p < 0.01.
